# Biomedical Microscopic Imaging in Computational Intelligence Using Deep Learning Ensemble Convolution Learning-Based Feature Extraction and Classification

**DOI:** 10.1155/2022/3531308

**Published:** 2022-06-27

**Authors:** Tammineedi Venkata Satya Vivek, Ayesha Naureen, Mohd. Shaikhul Ashraf, Sanhita Manna, Ahmed Mateen Buttar, P. Muneeshwari, Mohd Wazih Ahmad

**Affiliations:** ^1^Department of CSE, Koneru Lakshmaiah Education Foundation, Vaddeswaram, Guntur, Andhra Pradesh, India; ^2^Department of Computer Science and Engineering, B V Raju Institute of Technology, Narsapur, Medak Dist, Telangana 502313, India; ^3^Department of Botany, HKM Govt. Degree College Bandipora, Jammu and Kashmir, India; ^4^School of Technology, GITAM (Deemed to be University), Bangalore, Karnataka, India; ^5^Department of Computer Science, University of Agriculture Faisalabad, 38000, Pakistan; ^6^Institute of Artificial Intelligence and Machine Learning, Saveetha school of Engineering(SIMATS), Chennai, Tamilnadu, India; ^7^ASTU, Adama, Ethiopia

## Abstract

Microscopy image analysis gives quantitative support for enhancing the characterizations of various diseases, including breast cancer, lung cancer, and brain tumors. As a result, it is crucial in computer-assisted diagnosis and prognosis. Understanding the biological principles underlying these dynamic image sequences often necessitates precise analysis and statistical quantification, a major discipline issue. Deep learning methods are increasingly used in bioimage processing as they grow rapidly. This research proposes novel biomedical microscopic image analysis techniques using deep learning architectures based on feature extraction and classification. Here, the input image has been taken as microscopic image, and it has been processed and analyzed for noise removal, edge smoothening, and normalization. The processed image has been extracted based on their features in microscopic image analysis using ConVol_NN architecture with AlexNet model. Then, the features have been classified using ensemble of Inception-ResNet and VGG-16 (EN_InResNet_VGG-16) architectures. The experimental results show various dataset analyses in terms of accuracy of 98%, precision of 90%, computational time of 79%, SNR of 89%, and MSE of 62%.

## 1. Introduction

Advanced microscopy allows us to collect large numbers of time-lapse photos to see how tissues, cells, and molecules change over time. Microscopy images generally have a wide range of SNR and include a great deal of data, necessitating the use of several parameters and time-consuming iterative methods to analyze. However, manually processing picture data is inefficient or even impossible nowadays due to the vast volume of image data that continues to grow. Computerized approaches enhance efficiency and objectivity greatly, gaining a lot of attention in recent literature [1]. ML techniques are widely and successfully used in medicine as well as biology research [2]. ML gains expertise from data representations instead of nonlearning-based approaches that may not accurately transfer domain knowledge into rules. Traditional ML methods rarely interact directly with raw data instead of relying primarily on data representations, requiring extensive domain knowledge and complex engineering. The most general approaches for imaging living cells with transmitted light are phase contrast (PC) as well as differential interference contrast (DIC) [3]. They convert data encoded in imaging field's phase into final image's intensity distribution, whereas atomic force, as well as scanning electron microscopy, is better for rendering 3D quantitative shape measurements of samples. A recent trend in microscope development is super-resolution microscopy. It records biological events at the nanoscale scale, breaking the diffraction limit [4]. These new methods allow us to collect large amounts of high-quality photos containing various biomedical data. Simultaneously, we face unique obstacles in digesting these images through quantitative data processing. As a result, utilizing computational approaches to improve microscopy's performance as well as make it multifunctional in postprocessing has become a hot topic in field.

DL is a representation learning technique that takes raw data directly and learns representations automatically, and it may be utilized for object identification, image segmentation, and target classification. DL methods have advanced artificial intelligence significantly in recent years, and they have been effectively used in computer vision, NLP, speech recognition, medical imaging, computational biology, and other fields. It has placed first in various categories, including image classification and speech recognition has won multiple competitions in biomedical image study, including brain picture segmentation [5] and mitosis detection, by autonomously detecting hidden data structures. Meanwhile, it has shown great promise in various other biomedical applications. One of the primary advantages of DL over traditional image processing methods is that humans do not model its layers of characteristics. Instead, a general-purpose learning process is used to learn the attributes from the data. Furthermore, as computer science has progressed, DL can now deal with large specifications issues and improve speed, accuracy, and robustness in complex scenarios [6].

### 1.1. Research Contribution Is as Follows

- To design novel techniques in biomedical microscopic image analysis based on feature extraction and classification utilizing deep learning architectures
- To collect the microscopic image and process them for noise removal, edge smoothening, and normalization
- To extract the features using ConVol_NN architecture with AlexNet model
- To classify the extracted features using ensemble of Inception-ResNet and VGG-16 architectures
- The experimental results show various dataset analyses regarding accuracy, precision, computational time, SNR, and MSE

## 2. Related Works

DL has recently sparked a lot of interest in microscope image analysis. In cryo-EM pictures, cell and nuclei detection [7] and nanoparticle detection in SEM and TEM images [8] are two DL-based particle detection research types. DL methods outperform low-level image processing methods such as thresholding and others. In biomedical microscopy image analysis, the authors [9] surveyed prominent DNNs and summarised the findings for nuclei identification, cell segmentation, and tissue segmentation tasks. Many researchers utilize CNN to recognize cells in cryo-EM pictures and TEM images. Researchers looked at single-particle recognition on cryo-EM photos [10]. DeepEM is a software framework that they built. Their solution relies on a deep CNN to recognize cells in a noisy environment. DeepEM uses eight layers to extract features from an image, allowing it to work with fewer training photos. Square frames are used to detect cells in the provided image. DeepPicker [11] has taken a different approach to the same subject. DeepPicker is a CNN-based framework for fully automated particle selection from cryoEM pictures. Particle selection is mechanized by dividing it into 5 stages: scoring, cleaning, filtering, sorting, and iteration. This structure allowed for the verification of correctness between steps. The authors [12] focused on supervised learning in DNNs, particularly CNNs and RNNs and their applications in object detection, recognition, and NLP. The monograph [14] surveys general DL methods as well as their applications (primarily) in speech processing as well as computer vision. Book [13] established DL methods as well as gives speculative ideas for future research. The authors [15] examine several recent DL applications in medical image computing. The authors [16] discuss the use of DNNs in biomedical data processing, including omics, pictures, and signals. Many other studies introducing DL or associated topics exist due to advent of DL and its influences in a wide range of disciplines [17]. DNNs have been utilized to handle inverse problems in optical microscopy in certain recent studies [18]. DL was offered to improve optical microscopy spatial resolution [19]. DL was used to learn statistical changes through high degrees of abstraction to improve upon standard super-resolution techniques in fluorescence microscopy, according to [20]. DL has also been used to solve problems related to holographic picture reconstruction.

## 3. System Model

**Figure 1:**
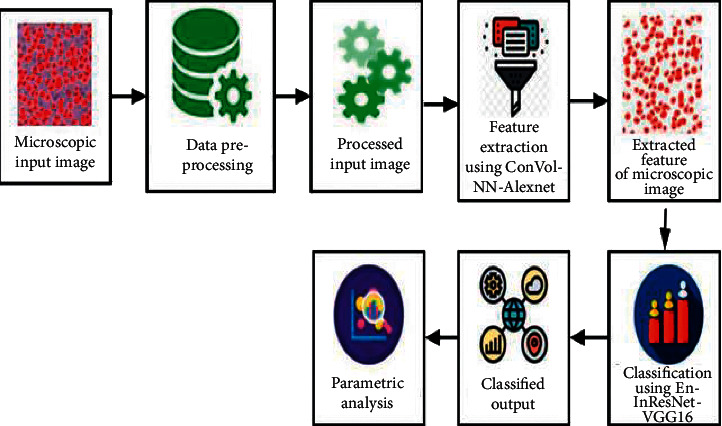
Overall proposed architecture.

This section proposes novel techniques in analysis of microscopic images based on feature extraction as well as classification utilizing DL methods. Initially, input has been processed for noise removal, edge smoothening, and normalization. The processed image has been extracted based on their features in microscopic image analysis using CNN architecture with AlexNet model. Then, the features were classified using ensemble of Inception-ResNet and VGG-16 architectures. The overall proposed architecture is shown in Figure 1.

In general, microscopic images are available as raw data for processing. As a result, the tiny images must be transformed into grayscale photographs. It is critical to reducing noise to enhance further processing effects, and noise removal is a common preprocessing step. A nonlinear filter called the median filter is used to eliminate noise in this work. Because it preserves the while reducing noise, it is commonly employed in image processing. It is done by slapping a 3 *∗* 3 mask over the image, calculating the median value, and then replacing the mask's center with a median filter. It is a noise reduction technique created to avoid over-amplification of noise. It only works on limited areas rather than the complete image.

### 3.1. ConVol_NN_AlexNet Model-Based Feature Extraction

**Figure 2:**
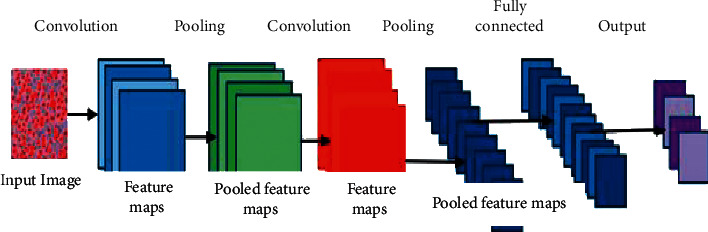
ConVol_NN architecture.

As shown in Figure 2, CNN comprises a convolutional, pooling, full connection, and an output layer. Each neuron in a regular NN must be connected to all neurons in preceding layer, resulting in a vast amount of calculation; each neuron in a CNN just pulls information from the previous layer's local perception, effectively reducing the number of specifications. Enhancing number of convolution kernels can help obtain additional features as well as increase model's expressive ability to some level.

The deconvolution layer, also known as transposed convolution, is commonly utilized in image restoration and super-resolution reconstruction to restore extracted feature image to original image. This study employs the deconvolution layer to classify images, then restores features recovered by convolution layer, minimizes number of feature maps over time, and finally outputs via the complete connection layers. The number of output characteristic graphs is considerably reduced due to the deconvolution operation compared to output of convolution layer, reducing number of nodes. Downsampling layer is another name for pooling layer [12]. There are three types of pooling: mean, maximal, and random pooling. Maximum pooling preserves image's texture data better, while mean pooling effectively preserves the image's background information, and random pooling is a mix of two, with matching probability assortment determined based on the element value of the sample region. The SoftMax classifier is typically used in the output layer to tackle multiclassification issues since evaluation amount is small and training pace is fast. Assume that function is given in the following way:

(1) $$\begin{array}{c} h_{\theta }\left(x^{\left(i\right)}\right)=\left[\begin{array}{c} p\left(y^{\left(i\right)}=1|x^{\left(i\right)};\theta \right)\\ p\left(y^{\left(i\right)}=2|x^{\left(i\right)};\theta \right)\\ \vdots \\ p\left(y^{\left(i\right)}=\text{K}|x^{\left(i\right)};\theta \right) \end{array}\right]\\ =\frac{1}{\sum_{\text{j}=1}^{K}e^{\theta_{j}^{r}x^{\left(i\right)}}}\left[\begin{array}{c} e^{\theta_{1}^{r}x^{\left(i\right)}}\\ e^{\theta_{2}^{r}x^{\left(i\right)}}\\ \vdots \\ e^{\theta_{k}^{r}x^{\left(i\right)}} \end{array}\right]. \end{array}$$

**Figure 3:**
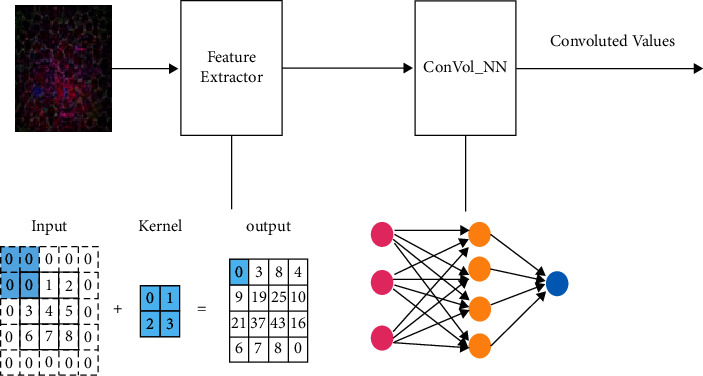
Architecture of ConVol_NN _AlexNet based on feature extraction.

Only the input and output layers are present in SoftMax's NNs. After adding hidden layers to the design, the concept of a deep neural layer arose. The network's numerous hidden layers helped the model learn faster. Method performs better when there are roughly 50 to 60 neurons in hidden layer. SoftMax loss is utilized in fully connected layer's output layer. ReLu is used as the activation function in hidden layers, while in the output layer, SoftMax is used as an activation function. Architecture of ConVol_NN_AlexNet based on feature extraction is shown in Figure 3.

The deconvolution operation minimizes number of feature maps, and output is then carried out over whole connection layer. Input of full connection layer is substantially minimized due to simplicity of feature maps. To limit overfitting degree of model, add a penalty component to loss function to prevent specifications from being too large or too small and keep method basic. After regularisation, the loss function is $\overset{`}{J}$, as shown in the following equation:

(2) $$\begin{array}{c} J^{\prime }\left(\theta \right)=J\left(\theta \right)+\alpha \Omega \left(\theta \right). \end{array}$$

L2 regularisation is utilized in this paper to improve model generalization, and method weight is reduced to near zero, as shown in the following equation:

(3) $$\begin{array}{c} J^{\prime }\left(\omega \right)=J\left(\omega \right)+\frac{1}{2}\alpha {\left\|\omega \right\|}_{2}^{2}. \end{array}$$

After the convolution layer, the batch normalizing (BN) layer is expanded to normalize the data, which, not only speeds up network convergence but also helps to overcome gradient disappearance as well as explosion issues among them, is sample mean, *µ* is sample variance *σ*, and *ε* is a constant close to 0.

(4) $$\begin{array}{c} {x^{\prime }}_{i}=\frac{x_{i}-\mu }{\sqrt{\sigma^{2}+\varepsilon }},\\ y_{i}=\gamma {x^{\prime }}_{i}+\beta ={\text{BN}}_{\gamma ,\beta }\left(x_{i}\right)\;w^{\prime }\\ =w-\alpha *\nabla \left(w;x^{\left(i\right)};y^{\left(i\right)}\right). \end{array}$$

### 3.2. Ensemble of Inception-ResNet and VGG-16 (EN_InResNet_VGG-16) Architectures in Classification

We execute convolutions with varying kernel sizes in parallel in each Inception module and then concatenate output from these parallel operations. Input is provided by the layer immediately preceding this block. This 1 × 1 Conv1D is a low-cost operation that acts as a dimensionality reduction layer for input features and is much easier to work with when extra channel is removed as seen in Figure 3. This 1 × 1 Conv1D bottleneck is referred to as a bottleneck because it lowers the number of input channels. The maximum kernel size hyperparameter determines the kernel sizes of 1 × 3 and 1 × 5 Conv1D layers.

**Figure 4:**
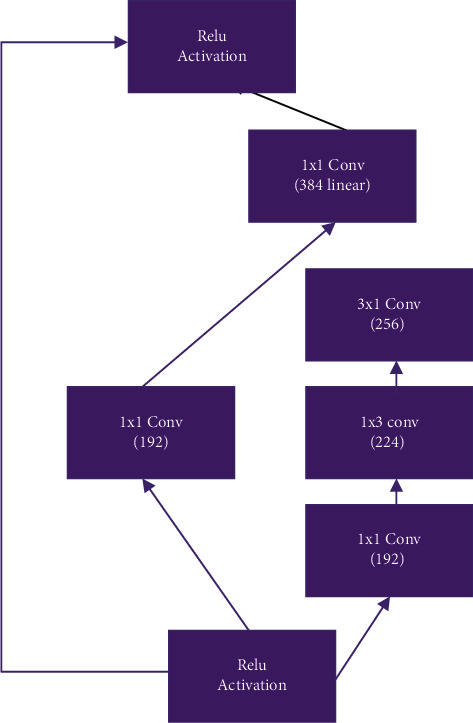
Inception-ResNet network intergrid models. (a) Inception-ResNet-A, (b) Inception-ResNet-B, and (c) Inception-Resnet-C block of the network.

**Figure 5:**
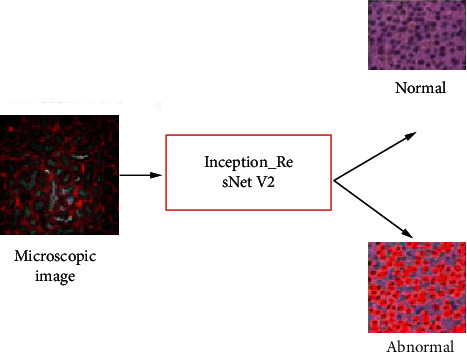
Inception ResNet architecture.

The stem layer is identical to that of the InceptionV4 model, while the remainder is made up of (a) 35 × 35 grid Inception ResNet-A module, (b) 35 × 35 to 17 × 17 Reduction-A module, (c) 17 × 17 grid Inception ResNet-B module, (d) 17 × 17 to 8 × 8 Reduction-B module, and (e) 8 × 8 grid InceptionResNet-C module [33]. Global average pooling was implemented instead of flatten to prevent overfitting in convolutional structure as illustrated in Figure 4 natively. Compared to the flatten approach, global average pooling is more parameter efficient [34]. After that, a dropout layer was added with a preset value of 0.8 as illustrated in Figure 5.

To boost computational speed, factorize 5 × 5 convolution into two 3 × 3 convolution processes. A 5 × 5 convolution is 2.78 times more classy than a 3 × 3 convolution, which may appear paradoxical. As a result, stacking two 3 × 3 convolutions improves performance. Furthermore, they factorize *n* × *n* filter convolutions into a mix of 1 × *n* and *n* × 1 convolutions. A 3 × 3 convolution, for example, is the same as executing a 1 × 3 convolution first, then a 3 × 1 convolution on the output. Module's filter banks were enlarged to remove representational bottleneck. BatchNorm after summation was not used in the original research to train classical on a single dataset. As a result, Inception-ResNet models could obtain greater accuracies at a lower epoch.

Finally, SoftMax activation function *σ* was utilized in dense layer, as stated in equation (5), where *x* and *y* denote input and output, *K* denotes number of classes, and *e* is usual exponential function, that is, *e* *≈* 2.718.

(5) $$\begin{array}{c} w^{\prime }=w-\alpha *\nabla \left(w;x^{\left(i\right)};y^{\left(i\right)}\right). \end{array}$$

The iterative SGD technique was used for backpropagation optimization, as shown in equation (5), where *w* relates weight, *α* relates learning rate, and ∇ (*w*; *x* (*i*); *y* (*i*)) relates gradient to weight, input, and output/label.  Table 1 shows full list of configurable hyperparameter parameters. These hyperparameters, such as decay and momentum, were later fine-tuned to improve accuracy.

All network layers used 3 × 3 filters with a max-pooling size of 2 and a stride and pad size of 1. Figure 2 shows the VGG-16 architectural block diagram, consisting of 16 layers, including 13 convolution layers, ReLu layers, five max-pooling layers, and three fully connected layers with SoftMax layer.  Table 2 shows VGG-16's architecture, including 13 convolutional layers and three fully connected layers. VGG-16's default input image size is 224 × 224 pixels. Size of feature map is cut in half after each pooling layer. For example, before fully connected layers 7 × 7, last feature map has 512 channels and is enlarged into a vector with 25,088 (7 × 7 × 512) channels.

**Figure 6:**
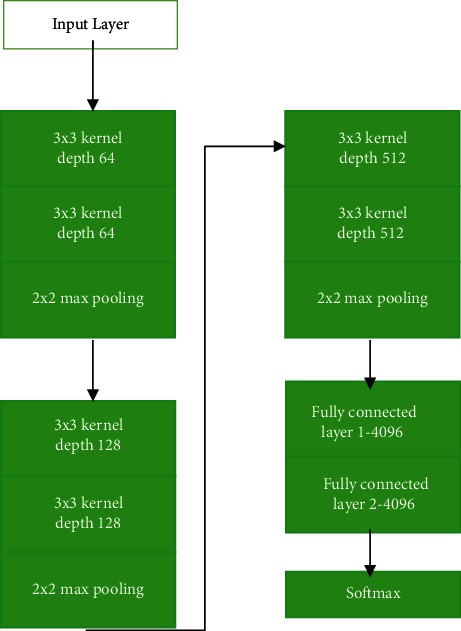
VGG-16 model architecture–13 convolutional layers and 2 fully connected layers and 1 SoftMax classifier VGG-16.

The VGG-16 network, as depicted in Figure 6, has following structure: first and second convolutional layers are made up of 64 3 × 3 feature kernel filters. As the input image goes through first and second convolutional layers, dimensions change to 224 × 224 × 64. The filter size of 124 feature kernel filters in the third and fourth convolutional layers is 3 × 3. In fifth, sixth, and seventh levels, convolutional layers with a kernel size of 3 × 3 are used. There are two sets of convolutional layers, one with a kernel size of 3 × 3. A maximum pooling layer with a stride of 1 follows these layers. Finally, 14th and 15th levels are 4096-unit fully connected hidden layers, with a 1000-unit SoftMax output layer following.

Using (1 × 1) convolutional layers enhances the nonlinearity of decision function without changing the convolutional layers' receptive fields. Moreover, to boost network width and flexibility to varied sizes, different convolutional kernel sizes are utilized for feature extraction and connection following 3 × 3 maximum pooling, and a 1 × 1 convolution is introduced.

## 4. Performance Analysis

In a simulated scenario, our system is compared against benchmarks for continuous variable mapping. Simulations were run in MATLAB software with a system having a 1.8 GHz Intel i7 processor and 16 GB of RAM. The effectiveness of the feature extraction and classification system is assessed using conventional and well-known metrics, allowing the system to be compared to other systems in the literature. A suitable assessment metric is influenced by several aspects, including the system's functionality. Among other things, these metrics can be used to assess computational complexity, processing time, memory use, and accuracy. In terms of accuracy, precision, computational time, MSE, and SNR, various performance metrics are provided below that can be utilized to calculate feature extraction and classification efficacy of DL methods.

### 4.1. Dataset Description

#### 4.1.1. PMID2019 Dataset

PMID2019 is the first phytoplankton detection dataset with high-resolution color photos. Each image has a resolution of 2040 1536 pixels, which is significantly greater than the photos in the comparison datasets. Microscopes are used to acquire photographs of phytoplankton in a laboratory setting. Each object in the images is given a bounding box and a ground-truth category by hand.

#### 4.1.2. CEM500K Dataset

We created CEMraw, an unlabeled cellular EM dataset that includes photos from 101 unrelated scientific investigations. Picture data superset, which provides for 591 3D image volumes and 9,626 2D images, was compiled using data from our studies as well as data from publicly available sources. We design a pipeline after obtaining this collection of heterogeneous photographs in which we first remove numerous almost identical images before filtering out low-quality and low data images. This yields a 25 GB 2D picture dataset with 0.5 × 10^6^ highly data-rich, relevant, and nonredundant.

#### 4.1.3. BSST265 Dataset

It comprises 79 fluorescence photos of antibody and DAPI labelled samples with a total of 7813 nuclei. One Schwann cell stroma-rich tissue cryosection, seven neuroblastoma patients, one Wilms patient, two NB cell lines, and a human keratinocyte cell line were used to create the photos. Furthermore, the dataset is diverse in preparation, imaging modality, magnification, SNR, and other technical characteristics. Type of BSST265 dataset preparation has been summarised based on the suitable composition of parameters in terms of diagnosis, magnification, SNR, and modality.

The various stages of microscopic image processing are shown in Table 3. Here, the various dataset input has been processed using proposed feature extraction as well as classification technique. Based on this processing of image, parametric analysis comparison has been carried out between proposed and existing techniques.

The above Table 4 shows comparative analysis for various microscopic image datasets between proposed and existing techniques. Here, the existing technique compared is CNN and DNN in terms of accuracy, precision, computational time, SNR, and MSE in calculating the error and loss function of the processed and classified image.

**Figure 7:**
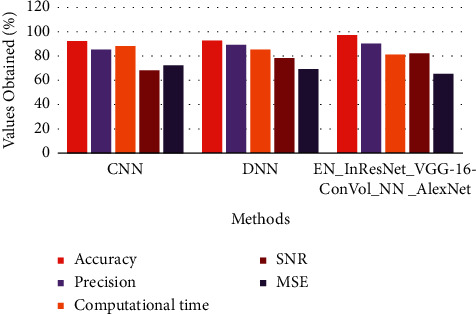
Comparative analysis of PMID2019 dataset in terms of accuracy, precision, computational time, SNR, and MSE.

**Figure 8:**
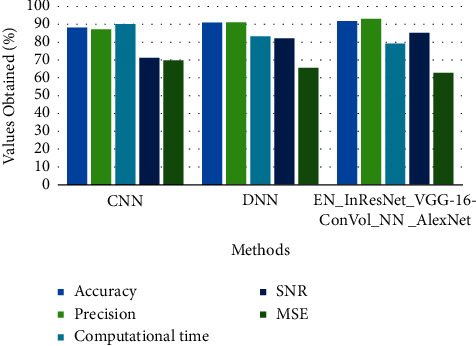
Comparative analysis of CEM500K dataset in terms of accuracy, precision, computational time, SNR, and MSE.

**Figure 9:**
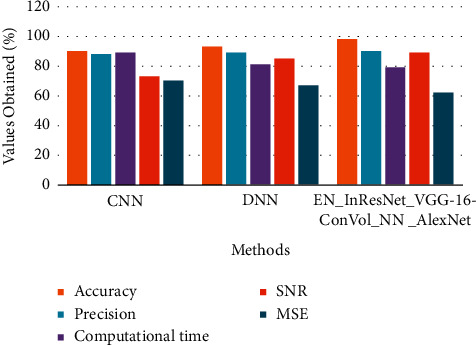
Comparative analysis of BSST265 dataset in terms of accuracy, precision, computational time, SNR, and MSE.

The above Figures 7–9 show comparative analysis of proposed and existing techniques in microscopic image analysis for various datasets. Here, the datasets compared are PMID2019, CEM500K, and BSST265 for proposed and existing techniques. For PMID2019 dataset, the proposed technique obtained accuracy of 97%, precision of 90%, computational time of 81%, SNR of 82%, and MSE of 65%; by analysis of CEM500K dataset, the proposed technique obtained accuracy of 92%, precision of 93%, computational time of 79%, SNR of 85%, and MSE of 63%; and BSST265 dataset in analysis of microscopic image, the proposed technique obtained accuracy of 98%, precision of 90%, computational time of 79%, SNR of 89%, and MSE of 62%. From this analysis of various datasets, the proposed technique obtained optimal results in microscopic image classification and feature extraction by validated training set and testing set of data.

## 5. Conclusion

This research proposes novel techniques in analysis of microscopic images based on feature extraction as well as classification utilizing DL methods. Initially, input has been processed for noise removal, edge smoothening, and normalization. The processed image has been extracted based on their features in microscopic image analysis using CNN architecture with AlexNet model. Then, the features were classified using ensemble of Inception-ResNet and VGG-16 architectures. The experimental results show various dataset analysis in terms of accuracy of 98%, precision of 90%, computational time of 79%, SNR of 89%, and MSE of 62%. From this analysis of various datasets, the proposed technique obtained optimal results in microscopic image classification and feature extraction by validated training set and testing set of data.

## Figures and Tables

**Table 1:** Default hyperparameter settings.

Number of epoch(s)	:	20
Train, val., test split	:	70 : 15 : 15
Number of batches (s)	:	32
Learning rate	:	1*e* − 3
Momentum	:	0.5
Decay	:	1*e* − 6
Activation	:	SoftMax
Optimizer	:	SGD

**Table 2:** Architecture of VGG-16 network.

Layer	Input size	Patch size
conv × 2	3 × 224 × 224	3 × 3/1
conv × 2	64 × 112 × 112	3 × 3/1
pool	64 × 224 × 224	2 × 2
conv × 3	128 × 56 × 56	3 × 3/1
pool	128 × 112 × 112	2 × 2
conv × 3	256 × 28 × 28	3 × 3/1
pool	256 × 56 × 56	2 × 2
conv × 3	512 × 14 × 14	3 × 3/1
pool	512 × 28 × 28	2 × 2
fc	25088	25088 × 4096
pool	512 × 14 × 14	2 × 2
fc	4096	4096 × 4096

**Table 3:** Processing of input microscopic image using proposed feature extraction and classification technique.

Dataset	Input microscopic image	Processed microscopic image	Extracted features of microscopic image	Classified microscopic image
PMID2019 dataset	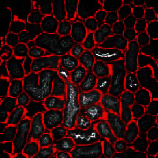	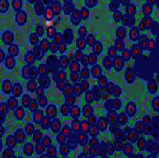	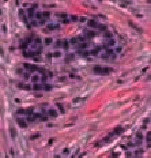	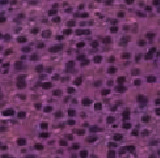
CEM500K dataset	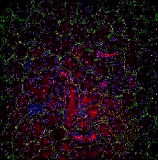	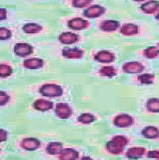	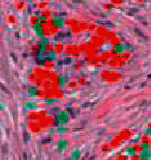	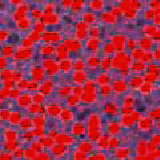
BSST265 dataset	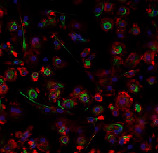	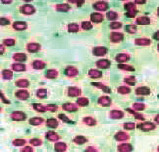	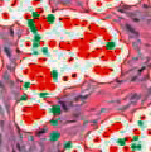	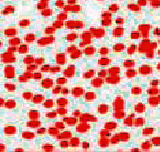

**Table 4:** Comparative analysis of various microscopic image datasets between proposed and existing techniques.

Datasets	Techniques	Accuracy	Precision	Computational time	SNR	MSE
PMID2019	CNN	92	85	88	68	72
	DNN	93	89	85	78	69
	EN_InResNet_VGG-16- ConVol_NN _AlexNet	97	90	81	82	65

CEM500K	CNN	88	87	90	71	70
	DNN	91	91	83	82	66
	EN_InResNet_VGG-16- ConVol_NN _AlexNet	92	93	79	85	63

BSST265	CNN	90	88	89	73	70
	DNN	93	89	81	85	67
	EN_InResNet_VGG-16- ConVol_NN _AlexNet	98	90	79	89	62

## Data Availability

All the data are available within the article.
